# Association between job insecurity and cardiovascular diseases in workers with type 2 diabetes mellitus

**DOI:** 10.5271/sjweh.4272

**Published:** 2026-05-01

**Authors:** Heejoo Park, Jian Lee, Youngsun Park, Juho Sim, Jin-Ha Yoon, Byungyoon Yun

**Affiliations:** 1 Department of Public Health, Graduate School, Yonsei University, Seoul, Republic of Korea.; 2 Department of Preventive Medicine, Yonsei University College of Medicine, Seoul, Republic of Korea.; 3 The Institute for Occupational Health, Yonsei University College of Medicine, Seoul, Republic of Korea.; 4 Institute for Innovation in Digital Healthcare, Yonsei University Health System.

**Keywords:** cohort study, economic impact, epidemiology, psychosocial stressors, unemployment, work productivity, work stress

## Abstract

**Objectives:**

This study analyzes the association between job insecurity, measured by cumulative unemployment, and the risk of cardiovascular disease (CVD) among middle-aged workers with type 2 diabetes mellitus.

**Methods:**

We utilized data from the National Health Insurance Service of Korea, focusing on patients with type 2 diabetes, aged 40–50 who were continuously employed in 2009–2010. Job insecurity was defined by cumulative unemployment in 2012–2016 and categorized as stable, partially stable, or unstable. Participants were followed until December 2023, with incident CVD as the primary outcome. Cox regression models estimated sex-stratified hazard ratios (HR) with 95% confidence intervals (CI), with additional subgroup and sensitivity analyses.

**Results:**

Among 128 704 participants (107 071 males and 21 633 females; median age 51 years), CVD occurred among 6.1% of males and 3.9% of females. Job insecurity was associated with an increased risk of CVD [males: HR 1.12 (95% CI 1.05–1.19) for partially stable, HR 1.25 (95% CI 1.16–1.34) for unstable; females: HR 1.00 (95% CI 0.85–1.19) for partially stable, HR 1.33 (95% CI 1.13–1.57) for unstable]. Subgroup analyses showed particularly elevated risks among low-income males and high-income females. By age, males aged 40–49 in the partially stable and unstable groups had increased CVD risks, while those aged 50–59 had the highest risk in the unstable group. Among females, significant associations appeared only in the 40–49 age group.

**Conclusions:**

Among middle-aged workers with type 2 diabetes, prolonged job insecurity was significantly associated with an increased risk of CVD.

Type 2 diabetes mellitus is a rapidly growing chronic disease worldwide and recognized as a major risk factor for cardiovascular disease (CVD) (1). According to the International Diabetes Foundation, the number of adults with type 2 diabetes mellitus worldwide is expected to reach approximately 783 million by 2045 (2). In Korea, the prevalence of type 2 diabetes has increased significantly from 7.6% in 2001 to 13.8% in 2018 and 13.9% in 2020 (3, 4). The increasing prevalence of type 2 diabetes has increased its economic burden worldwide, with costs projected to increase from USD 1.3 trillion in 2015 to USD2.1–2.5 trillion by 2030 (5). In Korea, the prevalence of type 2 diabetes in 2019 was 10.7%, contributing to an economic burden of USD 18.3 billion and a per capita cost of USD 4090 (6). This increase is largely driven by the steady increase in obesity and the adoption of westernized dietary habits (7).

Diabetics are particularly susceptible to the adverse effects of lifestyle and socioeconomic status-related factors as diabetes exacerbate their impact, further increasing susceptibility to CVD (8, 9). According to previous studies, CVD is influenced by various risk factors, including physical factor such as age, sex, genetic predisposition, as well as lifestyle factors, such as physical inactivity, smoking, and alcohol consumption (10–12). Furthermore, environmental and socioeconomic factors, including air pollution, noise exposure, work environment, and income, are also associated with heightened CVD risk (13, 14).

According to a previous study, the risk of myocardial infarction increases in the general population as the cumulative unemployment period lengthens (15). This association is largely attributed to chronic stress linked to prolonged unemployment, which over activates the hypothalamic-pituitary-adrenal (HPA) axis, disrupts neuroendocrine pathways, and induces chronic inflammation (16, 17). Chronic stress, characterized by prolonged cortisol elevation, also worsens insulin resistance, making diabetes management more challenging and accelerating disease progression (18). However, previous studies have primarily focused on the general population, overlooking the heightened vulnerability of high-risk groups such as individuals with type 2 diabetes. Considering that diabetes is a major risk factor for CVD, the added stress of job insecurity may further increase CVD risk among these individuals. To address this research gap, our study focuses on middle-aged paid workers with type 2 diabetes to investigate the combined impact of cumulative unemployment on the risk of CVD.

## Method

### Data source and study population

This study used data from the National Health Insurance Service (NHIS), which covers over 97% of the South Korean population (19). The NHIS database includes detailed demographic and socioeconomic information, such as employment status and industry sector, collected through structured lifestyle questionnaires. Additionally, the NHIS conducts biennial health examinations for adults, including various clinical and biochemical tests (20). This database also provides comprehensive records of outpatient visits, hospitalizations, diagnostic codes, medical procedures, and prescribed medications. The study population comprised workers aged 40–49 years and 50–59 years as of the index date (January 1, 2011) who had participated in a national health examination in 2009–2010. The index date was January 2011, with the participants’ employment status tracked in 2012–2016 to determine cumulative unemployment durations. Patients with type 2 diabetes were identified through prescriptions for antidiabetic medications associated with International Classification of Diseases, 10^th^ revision (ICD-10) codes E11–E14 (21).

The exclusion criteria were as follows: (i) missing information on socio-economic status, chronic disease factors, or lifestyle behaviors; (ii) participants employed in industrial sectors ‘T’ (activities of households as employers; undifferentiated goods- and services-producing activities of households for own use) or ‘U’ (activities of extraterritorial organizations and bodies); (iii) a previous history of CVD diagnosis or hospitalization due to CVD; (iv) occurrence of all-cause mortality or CVD within 1 year from the index date; (iv) implausibly long follow-up intervals; and (vi) missing employment insurance records in 2013–2016 (supplementary material, www.sjweh.fi/article/4272, table S1).

The Institutional Review Board (IRB) of Severance Hospital approved this study (IRB number: 4-2024-0615), which adhered to the ethical principles of the Declarations of Helsinki and Istanbul. The need for informed consent was waived owing to the retrospective nature of this study.

### Main outcomes and secondary outcomes

The primary outcome of this study was the incidence of CVD, defined as ≥3 hospitalizations and outpatient visits combined, associated with ICD-10 codes I21–I23, I50, or I63–I64 as identified through insurance claims data (22). Secondary outcomes included all-cause mortality, ischemic heart disease (IHD), ICD-10 codes I21–I23, heart failure (HF), ICD-10 code I50, and stroke, ICD-10 codes I63–I64,. The index date was 1 January 2011. Participants were followed until the occurrence of CVD, all-cause mortality, or 31 December 2023, whichever occurred first.

### Independent variable and covariates

Job insecurity, an independent variable, was defined as the cumulative unemployment period from 2011 to 2015, categorized into three groups based on employment status, which is derived from insurance type. The “stable” group comprised individuals who experienced no or <1 year of unemployment over the 5-year period. The “partially stable” group included individuals with 1–2 years of unemployment, while the “unstable” group included those unemployed for ≥3 years within the same period.

The analysis included the following covariates: age, household income quartile, residential area (Seoul, metropolitan, other), hypertension, dyslipidemia, duration of type 2 diabetes drug prescription, number of oral antidiabetic drugs taken last, type 2 diabetes complications, uncontrolled fasting blood sugar (FBS), fatty liver index (FLI), smoking history, alcohol consumption, and physical activity. Baseline covariates were assessed using insurance claims data and lifestyle questionnaires from a two-year look-back period prior to the index date 1 January 2011. Industrial clusters were classified into three industrial sectors based on the Korean Standard Industrial Classification (KSIC) and prior research: blue-collar (categories A-F, H), service (I, M, N, Q, R), and white-collar (G, J, K, L, O, P, S) (23) (supplementary table S1). Hypertension was defined as either a prescription for antihypertensive drugs according to ICD-10 codes I10–13, I15, or a systolic blood pressure (BP) of ≥140 mmHg or ≥90 mmHg (24). Dyslipidemia was identified through prescriptions for anti-dyslipidemia drugs corresponding to ICD-10 code E78 or total cholesterol ≥240 mg/dL (25). The duration of type 2 diabetes was categorized into <1 and ≥1 year. Oral antidiabetic drug use was categorized into four groups: 1, 2, >3 classes and insulin therapy. Type 2 diabetes-related complications were assessed based on the specificity of the ICD-10 codes related to type 2 diabetes (26). Uncontrolled FBS level was defined as ≥126 mg/dL. The FLI was categorized based on the standard threshold of FLI ≥30 (27). Individuals were classified as non-, ex-, and current smokers based on their smoking status using lifestyle questionnaires. Alcohol consumption was assessed by weekly intake levels, categorized as mild (<210 g for males and <140 g for females), moderate (<420 g for males and <350 g for females), and severe (≥420 g for males and ≥350 g for females) (28). The metabolic equivalent of the task (MET-minutes/week) for each participant’s physical activity was calculated based on their history of vigorous activity (7 MET), moderate activity (4 MET), and walking (2.9 MET) (29). Physical activity levels were further categorized into two groups: <600 MET-minutes per week and ≥600 or more MET-minutes per week (30).

### Statistical analysis

All the analyses were stratified by sex. Baseline characteristics were summarized using counts and percentages for categorical variables and medians with interquartile ranges (IQR) for continuous variables. Cumulative hazard curves for CVD by job insecurity groups were estimated using Cox proportional hazards models, with differences among groups assessed via log-rank tests. Differences in job insecurity experiences were examined using log-rank tests. Adjusted hazard ratios (HR_adj_) and 95% confidence intervals (CI) for the primary and secondary outcomes were estimated using multivariate Cox proportional hazards models. To validate the results, analyses included a crude model (unadjusted) and a final model adjusted for age, residential area, household income, industry sector, chronic disease-related factors, and lifestyle factors.

We conducted several sensitivity analyses. First, stratified analyses were conducted based on household age and income to assess the risk of CVD associated with job insecurity. Second, smoking intensity was further adjusted by incorporating smoking pack-years, calculated as the product of the number of cigarette packs smoked per day and the duration of smoking in years.

All statistical analyses were performed using SAS software version 8.2 (SAS Institute Inc, Cary, NC, USA) and R software version 4.0.3 (R Foundation for Statistical Computing, Vienna, Austria). Results were considered statistically significant at a two-sided P<0.05.

## Results

Following the recruitment of 146 175 individuals, 128 704 participants were included in the final analysis after applying the exclusion criteria (Supplementary figure S1). Table 1 presents the baseline characteristics of male participants by job insecurity, and table 2 presents those of female participants by job insecurity. Of these, 107 071 were males (83.2%) and 21 633 were females (16.8%). Among males, 68.6% (N=73 446) were in the stable group, 19.8% (N=21 163) in the partially stable group, and 11.6% (N=12 462) in the unstable group. Among females, 55.8% (N=12 065) were in the stable group, 24.9% (N=5388) in the partially stable group, and 19.3% (N=4180) in the unstable group.

**Table 1:** Baseline characteristics of **male** workers with type 2 diabetes by job insecurity status. [FBS=fasting blood sugar; GTP=glutamyl transpeptidase; IQR=interquartile range; MET=metabolic equivalent of the task]

		Men (N=107 071)							
		Stable (N=73 446)			Partially stable (N=21 163)			Unstable (N=12 462)	
		N (%)	Median (IQR)		N (%)	Median (IQR)		N (%)	Median (IQR)
Age			50.0 (46–54)			53.0 (48–56)			53.0 (48–57)
Household income									
	High	20 375 (27.7)			4711 (22.3)			2143 (17.2)	
	High–middle	19 830 (27.0)			4798 (22.7)			2517 (20.2)	
	Low–middle	17 565 (23.9)			5610 (26.5)			3443 (27.6)	
	Low	15 676 (21.3)			6044 (28.6)			4359 (35.0)	
Residential area									
	Seoul	13 537 (18.4)			4108 (19.4)			2359 (18.9)	
	Metropolitan	21 266 (29.0)			6016 (28.4)			3394 (27.2)	
	Others	38 643 (52.6)			11 039 (52.2)			6709 (53.8)	
Industrial sector									
	Blue-collar	44 690 (60.9)			12 722 (60.1)			7588 (60.9)	
	Service	9056 (12.3)			2564 (12.1)			1406 (11.3)	
	White-collar	19 700 (26.8)			5877 (27.8)			3468 (27.8)	
Hypertension									
	No	36 347 (49.5)			9751 (46.1)			5636 (45.2)	
	Yes	37 099 (50.5)			11 412 (53.9)			6826 (54.8)	
Dyslipidemia									
	No	44 843 (61.1)			13 217 (62.5)			7786 (62.5)	
	Yes	28 603 (38.9)			7946 (37.6)			4676 (37.5)	
Duration of diabetes (year)									
	<1	13 519 (18.4)			3735 (17.7)			2237 (18.0)	
	≥1	59 927 (81.6)			17 428 (82.4)			10 225 (82.1)	
Last class of oral antidiabetic drugs									
	1	32 694 (44.5)			9041 (42.7)			5219 (41.9)	
	2	31 724 (43.2)			9222 (43.6)			5483 (44.0)	
	≥3	5086 (6.9)			1666 (7.9)			922 (7.4)	
	Including insulin	3942 (5.3)			1234 (5.8)			838 (6.7)	
Diabetes complication									
	No	45 857 (62.4)			12 913 (61.0)			7592 (60.9)	
	Yes	27 589 (37.6)			8250 (39.0)			4870 (39.1)	
Uncontrolled FBS									
	No	27 237 (37.1)			7842 (37.1)			4491 (36.0)	
	Yes	46 209 (62.9)			13 321 (62.9)			7971 (64.0)	
Fatty liver index									
	<30	18 715 (25.5)			5480 (25.9)			3164 (25.4)	
	≥30	54 731 (74.5)			15 683 (74.1)			9298 (74.6)	
Smoking history									
	None	18 100 (24.6)			4978 (23.5)			2910 (23.4)	
	Ex–smoker	22 740 (31.0)			6335 (29.9)			3579 (28.7)	
	Current smoker	32 606 (44.4)			9850 (46.5)			5973 (47.9)	
Alcohol consumption									
	Mild	58 190 (79.2)			16 632 (78.6)			9677 (77.7)	
	Moderate	10 576 (14.4)			3119 (14.7)			1854 (14.9)	
	Severe	4680 (6.4)			1412 (6.7)			931 (7.5)	
Physical activity (MET–min/week)									
	0–<600	41 218 (56.1)			12120 (57.3)			7282 (58.4)	
	≥600	32 228 (43.9)			9043 (42.7)			5180 (41.6)	
Body mass index (kg/m^2^)			25.2 (23.4–27.2)			25.0 (23.2–27.0)			25.0 (23.2–27.1)
Waist circumference (cm)			87.0 (82.0–92.0)			86.0 (82.0–92.0)			87.0 (82.0–92.0)
Systolic blood pressure (mmHg)			127.0 (119.0–135.0)			128.0 (119.0–135.0.)			129.0 (119.0–135.8)
Diastolic blood pressure (mmHg)			80.0 (74.0–85.0)			80.0 (740–85.0)			80.0 (73.0–85.0)
Fasting blood glucose (mg/dL)			138.0 (115.0–174.0)			139.0 (115.0–177.0)			140.0 (116.0–182.0)
Gamma–GTP (IU/L)			45.0 (29.0–77.0)			46.0 (29.0–77.0)			46.0 (29.0–79.0)
Triglyceride (mg/dL)			162.0 (110.0–245.0)			162.0 (109.0–246.0)			164.0 (110.0–248.0)

**Table 2:** Baseline characteristics of **female** workers with type 2 diabetes, by job insecurity status. [FBS=fasting blood sugar; GTP=glutamyl transpeptidase; IQR=interquartile range; MET=metabolic equivalent of the task]

		Women (N=21 633)							
		Stable (N=12 065)			Partially stable (N=5 388)			Unstable (N=4180)	
		N (%)	Median (IQR)		N (%)	Median (IQR)		N (%)	Median (IQR)
Age			51.0 (47–55)			53.0 (48–56)			53.0 (49–57)
Household income									
	High	3402 (28.2)			1150 (21.3)			876 (21.0)	
	High–middle	3043 (25.2)			1418 (26.3)			960 (23.0)	
	Low–middle	2995 (24.8)			1452 (27.0)			1152 (27.6)	
	Low	2625 (21.8)			1368 (25.4)			1192 (28.5)	
Residential area									
	Seoul	2202 (18.3)			929 (17.2)			689 (16.5)	
	Metropolitan	3092 (25.6)			1406 (26.1)			1099 (26.3)	
	Others	6771 (56.1)			3053 (56.7)			2392 (57.2)	
Industrial sector									
	Blue-collar	4035 (33.4)			1881 (34.9)			1577 (37.7)	
	Service	3931 (32.6)			1897 (35.2)			1365 (32.7)	
	White-collar	4099 (34.0)			1610 (29.9)			1238 (29.6)	
Hypertension									
	No	6396 (53.0)			2723 (50.5)			2073 (49.6)	
	Yes	5669 (47.0)			2665 (49.5)			2107 (50.4)	
Dyslipidemia									
	No	7126 (59.1)			3145 (58.4)			2381 (57.0)	
	Yes	4939 (40.9)			2243 (41.6)			1799 (43.0)	
Duration of diabetes (year)									
	<1	2263 (18.8)			964 (17.9)			742 (17.8)	
	≥1	9802 (81.2)			4424 (82.1)			3438 (82.3)	
Last class of oral antidiabetic drugs									
	1	5586 (46.3)			2404 (44.6)			1820 (43.5)	
	2	4931 (40.9)			2248 (41.7)			1729 (41.4)	
	≥3	868 (7.2)			420 (7.8)			363 (8.7)	
	Including insulin	680 (5.6)			316 (5.9)			268 (6.4)	
Diabetes complication									
	No	7371 (61.1)			3318 (61.6)			2413 (57.7)	
	Yes	4694 (38.9)			2070 (38.4)			1767 (42.3)	
Uncontrolled FBS									
	No	4994 (41.4)			2281 (42.3)			1718 (41.1)	
	Yes	7071 (58.6)			3107 (57.7)			2462 (58.9)	
Fatty liver index									
	<30	6967 (57.8)			3061 (56.8)			2270 (54.3)	
	≥30	5098 (42.3)			2327 (43.2)			1910 (45.7)	
Smoking history									
	None	11 778 (97.6)			5217 (96.8)			4003 (95.8)	
	Ex–smoker	103 (0.9)			56 (1.0)			60 (1.4)	
	Current smoker	184 (1.5)			115 (2.1)			117 (2.8)	
Alcohol consumption									
	Mild	11 816 (97.9)			5244 (97.3)			4056 (97.0)	
	Moderate	223 (1.9)			120 (2.2)			108 (2.6)	
	Severe	26 (0.2)			24 (0.5)			16 (0.4)	
Physical activity (MET–min/week)									
	0–<600	7647 (63.4)			3488 (64.7)			2752 (65.8)	
	≥600	4418 (36.6)			1900 (35.3)			1428 (34.2)	
Body mass index (kg/m^2^)			24.7 (22.7–27.1)			24.8 (22.7–27.2)			24.9 (22.8–27.4)
Waist circumference (cm)			80.0 (75.0–86.0)			80.0 (75.0–86.0)			81.0 (75.0–87.0)
Systolic blood pressure (mmHg)			123.0 (115.0–132.0)			125.0 (115.0–134.0)			124.0 (116.0–135.0)
Diastolic blood pressure (mmHg)			79.0 (70.0–82.0)			80.0 (70.0–82.0)			80.0 (70.0–82.0)
Fasting blood glucose (mg/dL)			133.0 (111.0–166.0)			133.0 (111.0–167.0)			135.0 (112.0–169.0)
Gamma–GTP (IU/L)			23.0 (16.0–35.0)			23.0 (17.0–35.0)			23.0 (17.0–36.0)
Triglyceride (mg/dL)			124.0 (86.0–181.0)			125.0 (87.0–182.0)			129.0 (90.0–187.0)

Age was calculated as of the index date (1 January 2011). The median age was 51 years (IQR: 46–55 years) for males and 52 years (IQR: 47–56 years) for females. Stable workers were somewhat younger than those in the partially stable and unstable groups. For males, the unstable group had a higher prevalence of low household income, residence in non-metropolitan areas, blue-collar occupations, hypertension, absence of dyslipidemia, over one year of oral antidiabetic medication use, use of a single type of diabetes medication, absence of diabetes complications, status as current smoker, moderate alcohol consumption, and insufficient physical activity (<600 minutes/week). For females, the unstable group had a higher proportion with low household income, blue-collar employment, no diabetes complications, FLI scores <30, and non-smokers.

**Figure 1:**
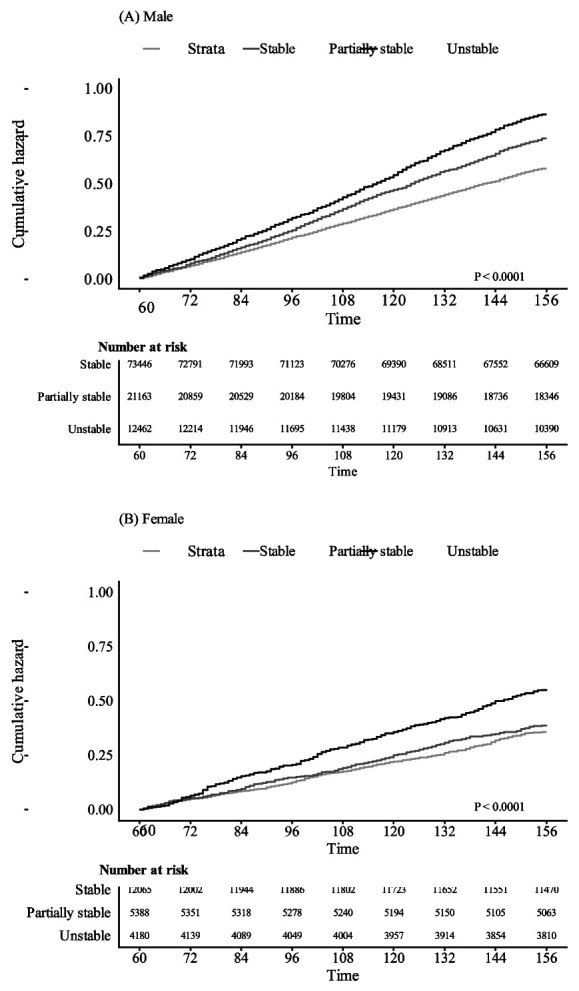
Cumulative incidence of CVD by employment status changes.

During a mean follow-up period of 12.6 years, 6535 males (6.1%) and 848 females (3.9%) experienced incident CVD. In the unstable group, 990 males (7.9%) and 222 females (5.3%) experienced CVD, compared with 1464 males (6.9%) and 204 females (3.8%) in the partially stable group, and 4081 males (5.6%) and 422 females (3.5%) in the stable group. The 5-year cumulative incidence of CVD after the index date (January 2011) was 3.8%, 4.6%, and 5.2% for the stable, partially stable, and unstable groups in males; and 2.2%, 2.5%, and 3.4% in females, respectively. The differences across employment stability groups were statistically significant in both men (P=2.0 × 10^−16^) and women (P=5.0 × 10^−7^) (figure 1).

Table 3 shows the results of the univariate and multivariate Cox regression models that assessed the association between job insecurity and CVD. Among males, the partially stable and unstable groups showed significantly elevated risk of CVD compared with the stable group (HR_adj_ 1.12, 95% CI 1.05–1.19 and HR_adj_1.25, 95% CI 1.16–1.34, respectively). Among females, only the unstable group showed a significantly increased risk (HR_adj_ 1.33, 95% CI 1.13–1.57), while the partially stable group did not differ significantly from the stable group (HR_adj_ aHR 1.00, 95% CI 0.85–1.19) (table 3).

**Table 3:** Adjusted hazard ratios (HR) and 95% confidence intervals (CI) of cardiovascular diseases by employment status.

Outcome	Sex	Job insecurity	N at risk	N of Events	Rate ^a^	Crude Model		Final Model ^b^
						HR (95% CI		HR (95% CI
Cardiovascular disease ^c^	Male	Stable	73 446	4081	439.5	Reference (1.00)		Reference (1.00)
		Partially stable	21 163	1464	554.4	1.27 (1.2–1.35)		1.12 (1.05–1.19)
		Unstable	12 462	990	644.3	1.49 (1.39–1.6)		1.25 (1.16–1.34)
	Female	Stable	12 065	422	272.9	Reference (1.00)		Reference (1.00)
		Partially stable	5388	204	296.6	1.09 (0.92–1.29)		1.00 (0.85–1.19)
		Unstable	4180	222	419.8	1.55 (1.32–1.82)		1.33 (1.13–1.57)

^a^ Rate were expressed per 100,000 person–years. ^b^ Final model was adjusted for adjusted for age, residential area, household income, Industrial Sector, hypertension, dyslipidemia, duration of diabetes, last class of oral antidiabetic drugs, uncontrolled fasting blood sugar, fatty liver index, smoking status, alcohol consumption, and physical activity. ^c^ CVD was defined as ≥3 combined hospitalizations or outpatient visits associated with ICD-10 codes I21–I23, I50, or I63–I64.

For both males and females, the risk of all-cause mortality significantly increased as job insecurity increased. The HF showed a pattern similar to that of the primary outcome, with a significant increase in risk among males as job insecurity increased. For females, a significant association between job insecurity and HF was observed only in the unstable group. For IHD, the risk significantly increased in the unstable group for males, whereas no significant increase was observed among females. For stroke, the risk significantly increased with cumulative unstable employment status among males, whereas it remained non-significant among females (supplementary table S2).

According to the analyses stratified by household age and income (table 4), the association between job insecurity and the risk of CVD was consistent across all categories. A significant association between job insecurity and an increased risk of CVD was observed among males in the 50–59 years age group (HR_adj_ 1.92, 95% CI 1.39–2.66) and in females in the 40–49 years age group (HR_adj_ 1.24, 95% CI 1.15–1.35). A significant association between job insecurity and an increased risk of CVD was observed in males in the low-income group (HR_adj_ 1.31, 95% CI 1.20–1.42) and in females in the high-income group (HR_adj_ 1.46, 95% CI 1.14–1.87).

**Table 4:** The association between job insecurity and the risk of cardiovascular diseases ^a^ stratified by age and income. All models were adjusted for age, residential area, household income, industrial sector, hypertension, dyslipidemia, duration of diabetes, oral antidiabetic drug, uncontrolled fasting blood sugar, fatty liver index, smoking status, alcohol consumption, and physical activity. [HR=hazard ratio; CI=confidence interval]

Variable		Sex	Job insecurity	At risk (N)	Events (N)	Rate ^b^	Adjusted HR (95% CI)
Age (years)	40–49	Male	Stable	33 628	1505	351.4	Reference (1.00)
			Partially stable	6396	366	454.5	1.16 (1.03–1.30)
			Unstable	3693	230	498.5	1.24 (1.08–1.43)
		Female	Stable	39 818	2576	514.9	Reference (1.00)
			Partially stable	14767	1098	598.3	1.10 (1.02–1.18)
			Unstable	8769	760	706.8	1.24 (1.15–1.35)
	50–59	Male	Stable	4971	120	187.4	Reference (1.00)
			Partially stable	1733	47	211.2	1.09 (0.77–1.53)
			Unstable	1157	55	374.1	1.92 (1.39–2.66)
		Female	Stable	7094	302	333.4	Reference (1.00)
			Partially stable	3655	157	337.5	0.96 (0.79–1.17)
			Unstable	3023	167	437.4	1.18 (0.98–1.43)
Income	High	Male	Stable	40 205	1849	361.8	Reference (1.00)
			Partially stable	9509	565	472.2	1.14 (1.03–1.33)
			Unstable	4660	296	509.0	1.17 (1.03–1.33)
		Female	Stable	6445	202	244.1	Reference (1.00)
			Partially stable	2568	89	271.2	1.03 (0.80–1.32)
			Unstable	1836	97	417.7	1.46 (1.14–1.87)
	Low	Male	Stable	33 241	2232	534.6	Reference (1.00)
			Partially stable	11 654	899	622.6	1.11 (1.03–1.20)
			Unstable	7802	694	726.7	1.31 (1.20–1.42)
		Female	Stable	5620	220	306.2	Reference (1.00)
			Partially stable	2820	115	319.8	0.98 (0.79–1.23)
			Unstable	2344	125	421.4	1.25 (1.00–1.56)

^a^ CVD was defined as ≥3 combined hospitalizations or outpatient visits associated with ICD-10 codes I21–I23, I50, or I63–I64. ^b^ Rate were expressed per 100,000 person–years.

Supplementary table S3 presents that adjusting for smoking pack-years, alone or with smoking status, did not alter the association between job insecurity and CVD risk. After adjusting for smoking pack-years and smoking status, males in the partially stable (HR_adj_ 1.11, 95% CI 1.05–1.18) and unstable groups (HR_adj_ 1.24, 95% CI 1.15–1.33) had higher CVD risk than the stable group. Among females, only the unstable group showed increased risk (HR_adj_ 1.34, 95% CI 1.13–1.58).

## Discussion

Our study identified a significant association between job insecurity, measured by cumulative unemployment duration, and the risk of CVD among patients with type 2 diabetes. The risk of CVD increased with greater job insecurity in both sexes, demonstrating a dose–response relationship, where higher levels of job insecurity were associated with a progressively greater risk. For males, significant associations were observed in both the partially stable and unstable groups, whereas for females, the association was significant only in the unstable group. Most secondary outcomes exhibited trends similar to those observed for CVD, showing increased risks with greater job insecurity.

One French study reported a significant association between the duration of past unemployment and the prevalence of non-fatal CVD events, such as myocardial infarction (OR_adj_ 1.75, 95% CI 1.12–2.74), after adjusting for demographic, lifestyle, and employment-related factors (15). A previous study conducted in Italy demonstrated that unemployed males had significantly higher risks of all-cause mortality (OR_adj_ 1.82, 95% CI 1.37–2.41) and coronary heart disease (OR_adi_ 2.58, 95% CI 1.05–6.37), whereas no such associations were observed in females (all-cause mortality OR_adj_ 1.29, 95% CI 0.87–1.92) (31). Our study revealed a significant positive association between prolonged unemployment duration and CVD incidence, in line with existing evidence. For all-cause mortality, risks were elevated in both partially stable and unstable groups for both sexes. For CVD, the risk was significant in both the partially stable and unstable groups for males, whereas it was significant only in the unstable group for females. According to social-role theory, these differences may arise from culturally defined gender roles, where men often face pressure as primary breadwinners and women navigate stress from balancing professional and caregiving responsibilities (32, 33). Such role-based expectations could explain the observed gender differences in responses to job insecurity.

In Anfossi et al's systematic review (34), six studies examined job insecurity as a risk factor for ischemic heart disease. While most studies showed a positive, but not statistically significant, association, the overall evidence from these general or occupationally heterogeneous populations was limited and inconsistent. In contrast, our investigation focused on individuals with type 2 diabetes—a population with elevated baseline cardiometabolic vulnerability. We found a robust and statistically significant association between job insecurity and adverse cardiovascular outcomes, suggesting that the detrimental effects of job insecurity on cardiovascular health may be amplified in high-risk clinical subgroups.

In age-stratified analyses, the highest risk was observed among males in their 50s in the unstable job group, while among females, the strongest association was found in the 40–49 age group. These age- and sex-specific patterns align with prior literature indicating that middle-aged men and women may face distinct stressors related to employment and social roles. Previous studies have noted that men in their 50s often experience increased financial pressures, while women in their 40s frequently manage both occupational and caregiving responsibilities, potentially contributing to differential vulnerability to job-related stress (35–37).

In a previous study (38), compared to low-income females, low-income males faced a higher risk of CVD owing to job insecurity. Similarly, in our study, males in the unstable employment group were particularly vulnerable and exhibited a higher risk of CVD in the low-income group. In contrast, females in the unstable employment group with high household income demonstrated a heightened risk of CVD. This disparity may stem from traditional gender roles. Males often shoulder financial responsibilities, leading to increased stress in low-income situations, while high-income females may face heightened stress from social expectations and professional demands (32, 33, 39). These findings underscore the importance of considering both socioeconomic and gender-specific factors when addressing the health impacts of job insecurity.

This study had several strengths. First, to our knowledge, this is the first study to demonstrate an association between job insecurity and the risk of CVD among individuals with type 2 diabetes in Asian countries. Second, it used a representative cohort comprising over 97% of the general population of the Republic of Korea (19), with comprehensive adjustment for a wide array of potential confounders, including socioeconomic status, physical activity, medication use, and lifestyle habits. Third, the long follow-up period allowed for the evaluation of longitudinal associations between job insecurity and cardiovascular outcomes, thereby enhancing the reliability of the observed effects over time.

However, our study had some limitations. First, due to the limited data available in the NHIS database, certain unmeasured confounding factors, such as dietary habits and genetic predispositions, could not be included in our analysis. Second, as this was an observational study, a definitive causal relationship between job insecurity and CVD could not be established. Third, residual confounding may persist due to limitations in capturing the complexity of smoking behavior. Specifically, our adjustments for smoking status and smoking pack-years may not fully account for time-varying changes in smoking behavior or inaccuracies in self-reported smoking data, potentially affecting the precision of our risk estimates. Fourth, the generalizability of the findings may be limited owing to differences in occupational and cultural backgrounds between countries. However, these results still offer valuable insights compared to other similar studies conducted in Asian countries, where socioeconomic and employment contexts might be more comparable to those in Korea. Fifth, owing to missing data on the cause of death, we could not analyze specific causes of mortality. Therefore, we focused on the association between job insecurity and overall health outcomes.

In summary, prolonged job insecurity demonstrates a gender difference in its impact on the risk of newly diagnosed CVD, particularly among individuals with type 2 diabetes. Targeted policies and comprehensive strategies addressing job insecurity and health management in individuals with type 2 diabetes are required to effectively prevent these outcomes.

## Supplementary material

Supplementary material

## References

[1] Kumar A, Gangwar R, Zargar AA, Kumar R, Sharma A. Prevalence of Diabetes in India: A Review of IDF Diabetes Atlas 10th Edition. Current diabetes reviews 2024; 20(1):e130423215752. 10.2174/1573399819666230413094200 37069712

[2] Sun H, Saeedi P, Karuranga S, Pinkepank M, Ogurtsova K, Duncan BB et al. IDF Diabetes Atlas: Global, regional and country-level diabetes prevalence estimates for 2021 and projections for 2045. Diabetes Res Clin Pract 2022 Jan;183:109119. 10.1016/j.diabres.2021.109119 34879977 PMC11057359

[3] Jeong D, Mok J, Jeon D, Kang HY, Kim HJ, Kim HS et al. Prevalence and associated factors of diabetes mellitus among patients with tuberculosis in South Korea from 2011 to 2018: a nationwide cohort study. BMJ Open 2023 Mar;13(3):e069642. 10.1136/bmjopen-2022-069642 36889835 PMC10008237

[4] Kim SM, Lee JS, Lee J, Na JK, Han JH, Yoon DK et al. Prevalence of diabetes and impaired fasting glucose in Korea: Korean National Health and Nutrition Survey 2001. Diabetes Care 2006 Feb;29(2):226–31. 10.2337/diacare.29.02.06.dc05-0481 16443864

[5] Bommer C, Sagalova V, Heesemann E, Manne-Goehler J, Atun R, Bärnighausen T et al. Global Economic Burden of Diabetes in Adults: projections From 2015 to 2030. Diabetes Care 2018 May;41(5):963–70. 10.2337/dc17-1962 29475843

[6] Oh SH, Ku H, Park KS. Prevalence and socioeconomic burden of diabetes mellitus in South Korean adults: a population-based study using administrative data. BMC Public Health 2021 Mar;21(1):548. 10.1186/s12889-021-10450-3 33743612 PMC7980668

[7] Yang HJ, Kim MJ, Hur HJ, Jang DJ, Lee BK, Kim MS et al. Inverse Association of the Adequacy and Balance Scores in the Modified Healthy Eating Index with Type 2 Diabetes in Women. Nutrients 2023 Apr;15(7):1741. 10.3390/nu15071741 37049581 PMC10097397

[8] Bertoluci MC, Rocha VZ. Cardiovascular risk assessment in patients with diabetes. Diabetol Metab Syndr 2017 Apr;9(1):25. 10.1186/s13098-017-0225-1 28435446 PMC5397821

[9] Williams ED, Tapp RJ, Magliano DJ, Shaw JE, Zimmet PZ, Oldenburg BF. Health behaviours, socioeconomic status and diabetes incidence: the Australian Diabetes Obesity and Lifestyle Study (AusDiab). Diabetologia 2010 Dec;53(12):2538–45. 10.1007/s00125-010-1888-4 20740271

[10] Jagannathan R, Patel SA, Ali MK, Narayan KM. Global updates on cardiovascular disease mortality trends and attribution of traditional risk factors. Curr Diab Rep 2019 Jun;19(7):44. 10.1007/s11892-019-1161-2 31222515

[11] Dahlöf B. Cardiovascular disease risk factors: epidemiology and risk assessment. Am J Cardiol 2010 Jan;105(1 Suppl):3A–9A. 10.1016/j.amjcard.2009.10.007 20102968

[12] Ding C, O’Neill D, Bell S, Stamatakis E, Britton A. Association of alcohol consumption with morbidity and mortality in patients with cardiovascular disease: original data and meta-analysis of 48,423 men and women. BMC Med 2021 Jul;19(1):167. 10.1186/s12916-021-02040-2 34311738 PMC8314518

[13] Münzel T, Hahad O, Sørensen M, Lelieveld J, Duerr GD, Nieuwenhuijsen M et al. Environmental risk factors and cardiovascular diseases: a comprehensive expert review. Cardiovasc Res 2022 Nov;118(14):2880–902. 10.1093/cvr/cvab316 34609502 PMC9648835

[14] Bhatnagar A. Environmental Determinants of Cardiovascular Disease. Circ Res 2017 Jul;121(2):162–80. Available from: http://dx.doi.org/doi:10.1161/CIRCRESAHA.117.306458 10.1161/CIRCRESAHA.117.306458 28684622 PMC5777598

[15] Sanchez Rico M, Plessz M, Airagnes G, Ribet C, Hoertel N, Goldberg M et al. Cardiovascular burden and unemployment: A retrospective study in a large population-based French cohort. PLoS One 2023 Jul;18(7):e0288747. 10.1371/journal.pone.0288747 37459323 PMC10351739

[16] Rogerson O, Wilding S, Prudenzi A, O’Connor DB. Effectiveness of stress management interventions to change cortisol levels: a systematic review and meta-analysis. Psychoneuroendocrinology 2024 Jan;159:106415. Available from: http://dx.doi.org/https://doi.org/10.1016/j.psyneuen.2023.106415 10.1016/j.psyneuen.2023.106415 37879237

[17] Lagraauw HM, Kuiper J, Bot I. Acute and chronic psychological stress as risk factors for cardiovascular disease: insights gained from epidemiological, clinical and experimental studies. Brain Behav Immun 2015 Nov;50:18–30. Available from: http://dx.doi.org/https://doi.org/10.1016/j.bbi.2015.08.007 10.1016/j.bbi.2015.08.007 26256574

[18] Gianotti L, Belcastro S, D’Agnano S, Tassone F. The Stress Axis in Obesity and Diabetes Mellitus: an Update. Endocrines 2021;2(3):334–47. 10.3390/endocrines2030031

[19] Song SO, Jung CH, Song YD, Park CY, Kwon HS, Cha BS et al. Background and data configuration process of a nationwide population-based study using the korean national health insurance system. Diabetes Metab J 2014 Oct;38(5):395–403. 10.4093/dmj.2014.38.5.395 25349827 PMC4209354

[20] Shin DW, Cho J, Park JH, Cho BJ, Medicine F. National General Health Screening Program in Korea: history, current status, and future direction. Precis Future Med 2022;6(1):9–31. 10.23838/pfm.2021.00135

[21] Cho Y, Kim B, Kwon HS, Han K, Kim MK. Diabetes severity and the risk of depression: A nationwide population-based study. J Affect Disord 2024 Apr;351:694–700. Available from: http://dx.doi.org/https://doi.org/10.1016/j.jad.2024.01.181 10.1016/j.jad.2024.01.181 38302066

[22] Park JH, Kim DH, Park YG, Kwon DY, Choi M, Jung JH et al. Association of Parkinson Disease With Risk of Cardiovascular Disease and All-Cause Mortality: A Nationwide, Population-Based Cohort Study. Circulation 2020 Apr;141(14):1205–7. Available from: http://dx.doi.org/doi:10.1161/CIRCULATIONAHA.119.044948 10.1161/CIRCULATIONAHA.119.044948 32250706

[23] Zaitsu M, Kato S, Kim Y, Takeuchi T, Sato Y, Kobayashi Y et al. Occupational Class and Risk of Cardiovascular Disease Incidence in Japan: Nationwide, Multicenter, Hospital-Based Case-Control Study. J Am Heart Assoc 2019 Mar;8(6):e011350. Available from: http://dx.doi.org/doi:10.1161/JAHA.118.011350 10.1161/JAHA.118.011350 30845875 PMC6475056

[24] Bae EH, Lim SY, Jung JH, Oh TR, Choi HS, Kim CS et al. Chronic Kidney Disease Risk of Isolated Systolic or Diastolic Hypertension in Young Adults: A Nationwide Sample Based-Cohort Study. J Am Heart Assoc 2021 Apr;10(7):e019764. 10.1161/JAHA.120.019764 33787312 PMC8174338

[25] Cho SM, Lee H, Lee HH, Baek J, Heo JE, Joo HJ et al.; Korean Society of Lipid and Atherosclerosis (KSoLA) Public Relations Committee. Dyslipidemia Fact Sheets in Korea 2020: an Analysis of Nationwide Population-based Data. J Lipid Atheroscler 2021 May;10(2):202–9. 10.12997/jla.2021.10.2.202 34095012 PMC8159761

[26] Vimont A, Béliard S, Valéro R, Leleu H, Durand-Zaleski I. Prognostic models for short-term annual risk of severe complications and mortality in patients living with type 2 diabetes using a national medical claim database. Diabetol Metab Syndr 2023 Jun;15(1):128. 10.1186/s13098-023-01105-x 37322499 PMC10268447

[27] Nivukoski U, Niemelä M, Bloigu A, Bloigu R, Aalto M, Laatikainen T et al. Combined effects of lifestyle risk factors on fatty liver index. BMC Gastroenterol 2020 Apr;20(1):109. 10.1186/s12876-020-01270-7 32293287 PMC7157978

[28] Kalligeros M, Vassilopoulos A, Vassilopoulos S, Victor DW, Mylonakis E, Noureddin M. Prevalence of Steatotic Liver Disease (MASLD, MetALD, and ALD) in the United States: NHANES 2017-2020. Clin Gastroenterol Hepatol 2024 Jun;22(6):1330–1332.e4. Available from: http://dx.doi.org/https://doi.org/10.1016/j.cgh.2023.11.003 .https://doi.org/10.1016/j.cgh.2023.11.003 10.1016/j.cgh.2023.11.003 37949334

[29] Jeong SW, Kim SH, Kang SH, Kim HJ, Yoon CH, Youn TJ et al. Mortality reduction with physical activity in patients with and without cardiovascular disease. Eur Heart J 2019 Nov;40(43):3547–55. 10.1093/eurheartj/ehz564 31504416 PMC6855138

[30] López-Bueno R, López-Sánchez GF, Smith L, Sundstrup E, Andersen LL, Casajús JA. Higher physical activity is associated with lower activity limitation: cross-sectional analyses among the Spanish working population. Sci Sports 2023;38(3):247–54. Available from: http://dx.doi.org/https://doi.org/10.1016/j.scispo.2021.12.010 10.1016/j.scispo.2021.12.010

[31] d’Errico A, Piccinelli C, Sebastiani G, Ricceri F, Sciannameo V, Demaria M et al. Unemployment and mortality in a large Italian cohort. J Public Health (Oxf) 2021 Jun;43(2):361–9. 10.1093/pubmed/fdz100 31740960

[32] Tattarini G, Grotti R. Gender roles and selection mechanisms across contexts: a comparative analysis of the relationship between unemployment, self-perceived health and gender. Sociol Health Illn 2022 Mar;44(3):641–62. 10.1111/1467-9566.13449 35218011

[33] Tattarini G, Grotti R. Gender roles and selection mechanisms across contexts: a comparative analysis of the relationship between unemployment, self-perceived health and gender. Sociol Health Illn 2022 Mar;44(3):641–62. Available from: http://dx.doi.org/https://doi.org/10.1111/1467-9566.13449 10.1111/1467-9566.13449 35218011

[34] Moretti Anfossi C, Ahumada Muñoz M, Tobar Fredes C, Pérez Rojas F, Ross J, Head J et al. Work Exposures and Development of Cardiovascular Diseases: A Systematic Review. Ann Work Expo Health 2022 Jul;66(6):698–713. 10.1093/annweh/wxac004 35237787 PMC9250287

[35] Ferrie JE, Kivimäki M, Shipley MJ, Davey Smith G, Virtanen M. Job insecurity and incident coronary heart disease: the Whitehall II prospective cohort study. Atherosclerosis 2013 Mar;227(1):178–81. 10.1016/j.atherosclerosis.2012.12.027 23332775 PMC3940189

[36] Payne S, Doyal L. Older women, work and health. Occup Med (Lond) 2010 May;60(3):172–7. 10.1093/occmed/kqq030 20423947

[37] El Khoudary SR, Aggarwal B, Beckie TM, Hodis HN, Johnson AE, Langer RD et al.; American Heart Association Prevention Science Committee of the Council on Epidemiology and Prevention; and Council on Cardiovascular and Stroke Nursing. Menopause Transition and Cardiovascular Disease Risk: Implications for Timing of Early Prevention: A Scientific Statement From the American Heart Association. Circulation 2020 Dec;142(25):e506–32. 10.1161/CIR.0000000000000912 33251828

[38] Kezios KL, Lu P, Calonico S, Al Hazzouri AZ. History of Low Hourly Wage and All-Cause Mortality Among Middle-aged Workers. JAMA 2023 Feb;329(7):561–73. 10.1001/jama.2023.0367 36809322 PMC9945122

[39] Rao AH. Gendered Interpretations of Job Loss and Subsequent Professional Pathways. Gend Soc 2021;35(6):884–909. 10.1177/08912432211046303

