# Supplementary material for: Association between job insecurity and cardiovascular diseases in workers with type 2 diabetes mellitus
Source: Scand J Work Environ Health. 2026 Apr 30;52(3):292–301. doi: 10.5271/sjweh.4272 (PMC13184974; doi:10.5271/sjweh.4272)

Association between job insecurity and cardiovascular diseases in workers with type 2 diabetes mellitus[1]

by Heejoo Park, BSc, Jian Lee, BSc, Youngsun Park, BSc, Juho Sim, PhD, Jin-Ha Yoon, MD, PhD, Byungyoon Yun, MD, PhD[2]

1. Supplementary material
2. Correspondence to: Byungyoon Yun, MD, PhD, Department of Preventive Medicine, Yonsei University College of Medicine, 50-1 Yonsei-ro, Seodaemun-gu, Seoul 03722, Republic of Korea. [E-mail: yby3721@yuhs.ac]

Supplementary Table S1. Classification of Economic Activities by Code

Code	Industry
A	Agriculture, forestry and fishing
B	Mining and quarrying
C	Manufacturing
D	Electricity, gas, steam and air conditioning supply
E	Water supply; sewage, waste management, materials recovery
F	Construction
G	Wholesale and retail trade
H	Transportation and storage
I	Accommodation and food service activities
J	Information and communication
K	Financial and insurance activities
L	Real estate activities
M	Professional, scientific and technical activities
N	Business facilities management and business support services; rental and leasing activities
O	Public administration and defense; compulsory social security
P	Education
Q	Human health and social work activities
R	Arts, sports and recreation related services
S	Membership organizations, repair and other personal services
T	Activities of households as employers; undifferentiated goods– and services–producing activities of households for own use
U	Activities of extraterritorial organizations and bodies

Based on the Korean Standard Industrial Classification [KSIC], managed by Statistics Korea. Codes A–U represent major divisions of industries, used for categorizing participants' employment sectors.

Supplementary Table S2. The risk of secondary outcome and all-cause mortality according to sex and job Insecurity

Outcome	Sex	Job insecurity	N at risk	N of Events	Rate§	Crude Model	Final Model
**All–Cause Mortality**	**Male**	Stable	73446	3158	334.4	Reference (1.00)	Reference (1.00)
		Partially stable	21163	1561	579.1	1.75 (1.64–1.86)	1.45 (1.37–1.55)
		Unstable	12462	1236	785.3	2.39 (2.24–2.55)	1.86 (1.74–1.99)
	**Female**	Stable	12065	216	138.2	Reference (1.00)	Reference (1.00)
		Partially stable	5388	144	207.0	1.50 (1.22–1.85)	1.36 (1.10–1.68)
		Unstable	4180	163	303.2	2.21 (1.80–2.71)	1.83 (1.49–2.25)
**Ischemic heart disease**	**Male**	Stable	73446	1031	109.6	Reference (1.00)	Reference (1.00)
		Partially stable	21163	332	123.7	1.14 (1.00–1.29)	1.05 (0.93–1.19)
		Unstable	12462	237	151.5	1.40 (1.21–1.61)	1.25 (1.09–1.45)
	**Female**	Stable	12065	51	32.7	Reference (1.00)	Reference (1.00)
		Partially stable	5388	31	44.6	1.37 (0.88–2.14)	1.22 (0.78–1.92)
		Unstable	4180	23	42.8	1.32 (0.81–2.16)	1.09 (0.66–1.80)
**Heart Failure**	**Male**	Stable	73446	1139	121.1	Reference (1.00)	Reference (1.00)
		Partially stable	21163	408	15229	1.27 (1.13–1.42)	1.12 (1.00–1.26)
		Unstable	12462	272	173.8	1.46 (1.28–1.66)	1.23 (1.08–1.41)
	**Female**	Stable	12065	149	95.7	Reference (1.00)	Reference (1.00)
		Partially stable	5388	63	90.9	0.95 (0.71–1.28)	0.87 (0.65–1.17)
		Unstable	4180	95	177.9	1.87 (1.45–2.42)	1.63 (1.25–2.12)
**Stroke**	**Male**	Stable	73446	2165	231.3	Reference (1.00)	Reference (1.00)
		Partially stable	21163	820	307.7	1.34 (1.24–1.45)	1.15 (1.06–1.25)
		Unstable	12462	541	348.2	1.53 (1.39–1.68)	1.24 (1.12–1.36)
	**Female**	Stable	12065	244	157.1	Reference (1.00)	Reference (1.00)
		Partially stable	5388	114	164.9	1.05 (0.84–1.31)	0.98 (0.78–1.22)
		Unstable	4180	114	213.9	1.37 (1.10–1.71)	1.18 (0.94–1.47)

Final model was adjusted for adjusted for age, residential area, household income, Industrial Sector, hypertension, dyslipidemia, duration of diabetes, last class of oral antidiabetic drugs, uncontrolled fasting blood sugar, fatty liver index, smoking status, alcohol consumption, and physical activity

Supplementary Table S3. Association between job insecurity and cardiovascular diseases by different adjustments for smoking

Outcome	Sex	Job insecurity	N at risk	N of Events	Rate§	Model† HR (95% CI)	Model‡ HR (95% CI)
**Cardiovascular Diseases**	**Male**	Stable	71339	3981	441.4	Reference (1.00)	Reference (1.00)
		Partially stable	20683	1429	553.7	1.12 (1.03–1.19)	1.11 (1.05–1.18)
		Unstable	12198	966	642.1	1.25 (1.17–1.35)	1.24 (1.15–1.33)
	**Female**	Stable	12030	420	272.4	Reference (1.00)	Reference (1.00)
		Partially stable	5366	204	297.8	1.01 (0.86–1.20)	1.01 (0.86–1.20)
		Unstable	4170	221	418.9	1.33 (1.13–1.57)	1.34 (1.13–1.58)

§ Rate were expressed per 100,000 person–years.

Model†: Adjusted for age, residential area, household income, industrial sector, hypertension, dyslipidemia, duration of diabetes, last class of oral antidiabetic drugs, uncontrolled fasting blood sugar, fatty liver index, pack-years, alcohol consumption, and physical activity.

Model‡: Adjusted for all variables in Model† plus smoking status.

Abbreviations: HR, hazard ratio; CI, confidence interval;

eFigure1. Flow chart of participant selection process

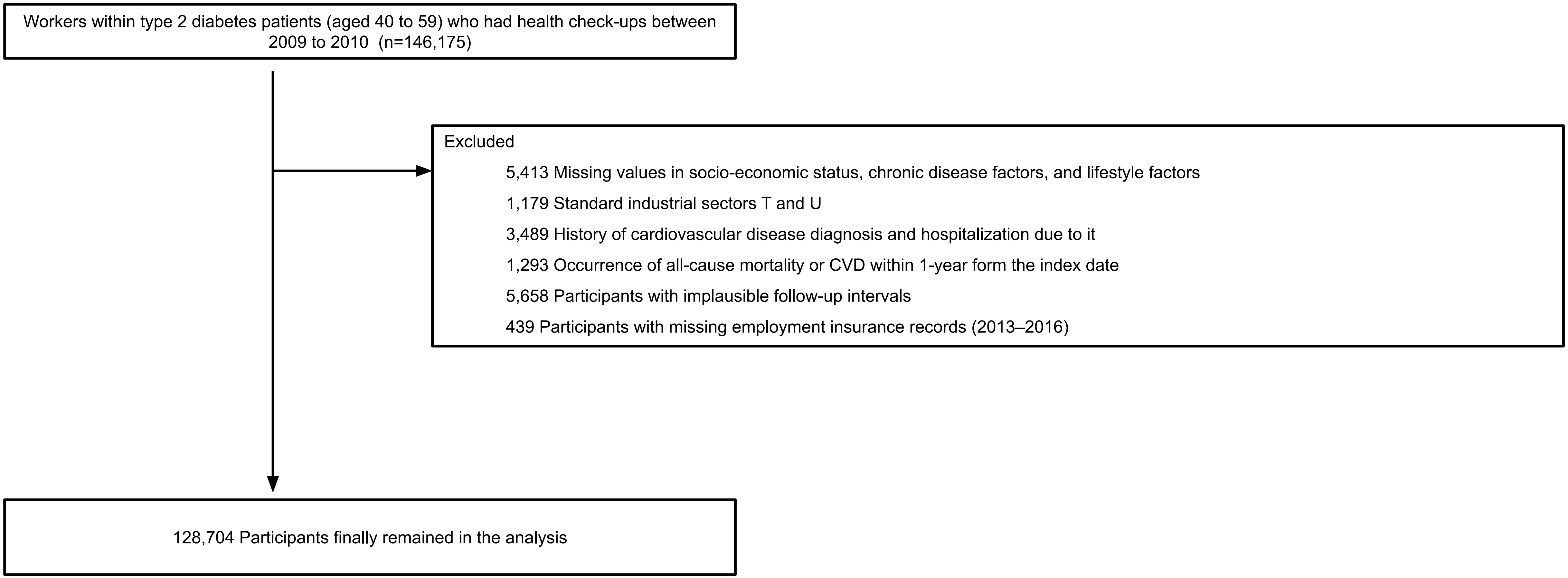

Supplement: Supplementary material [file SJWEH-52-292-S001.pdf]
